# Shared Decision-Making in Children’s Healthcare by Parents’ Immigrant Status: Findings from the 2021–2022 U.S. National Survey of Children’s Health

**DOI:** 10.1007/s10903-025-01771-1

**Published:** 2025-10-17

**Authors:** Sawsan Salah, Lori Anne Francis

**Affiliations:** Department of Biobehavioral Health, The Pennsylvania State University, University Park, PA USA

**Keywords:** Healthcare coordination, Health inequities, Parenting support

## Abstract

The increasing immigrant population in the U.S. faces unique challenges in navigating the healthcare system, including language and cultural barriers, and knowledge of services. Immigrant parents may face additional challenges as they attempt to navigate the healthcare system on behalf of their children. This study investigated associations between parents’ immigrant status and their perceptions of shared decision-making (SDM) in their child’s healthcare. Participants included 27,082 parent respondents from U.S. households in the 2021-22 National Survey of Children’s Health. Measures included parents’ immigrant status and reports of shared decision-making in their child’s healthcare. Potential moderators of the association between immigrant status and SDM included time spent providing or arranging children’s healthcare, household economic strain, parenting support, needing extra help to arrange care, and household language. Logistic regression analyses showed that compared to U.S.-born parents, immigrant parents were more likely to report lower levels of SDM. Immigrant parents who reported needing extra help coordinating or arranging healthcare services for their child had significantly higher odds of low SDM compared to their counterparts. To improve child health outcomes, immigrant families may benefit from increased SDM and support in coordinating their children’s healthcare services.

**Supplementary Information:**

The online version contains supplementary material available at 10.1007/s10903-025-01771-1.

## Background

Between 2022 and 2023, the immigrant population in the United States rose from 13.9% [1] to 14.8% [2], accounting for 47.8 million people in 2023; children comprised 6.0% of the immigrant population [2]. Because the U.S. is becoming increasingly diverse, it is important to consider the sociocultural factors affecting children’s access to healthcare [3, 4]. Huang et al. [5] found that U.S.-born children with immigrant parents utilize fewer healthcare services despite being in overall poorer health. This may occur because immigrant families face unique healthcare barriers, including language, and limited knowledge of available services, which may increase health disparities between children with immigrant parents and children with U.S.-born parents [3–6].

Shared decision-making (SDM) in healthcare is a process that involves at least one physician and one patient who discuss and implement an agreed-upon treatment plan [7, 8]. Research showed that SDM and effective patient-provider communication are crucial in influencing healthcare compliance [9–12]. Studies have also shown that SDM is associated with positive health outcomes and patient satisfaction – even among immigrant groups [11, 13]. Yet, disparities exist in SDM. Whites are more likely to engage in SDM than ethnic minority groups [10, 14, 15]. Moreover, ethnic minorities who have lower English proficiency are less likely to receive sufficient information regarding their medical needs and feel empathy from their providers [16–18]. For example, Hispanic and Asian patients report poor communication with their providers [19, 20]. Language barriers may amplify this issue, limiting opportunities for SDM and the quality of care received [21, 22]. While most studies on SDM and its effect on health outcomes were conducted on U.S.-born adults, little is known about SDM in immigrant populations and its impact on children’s health outcomes.

**Fig. 1:**
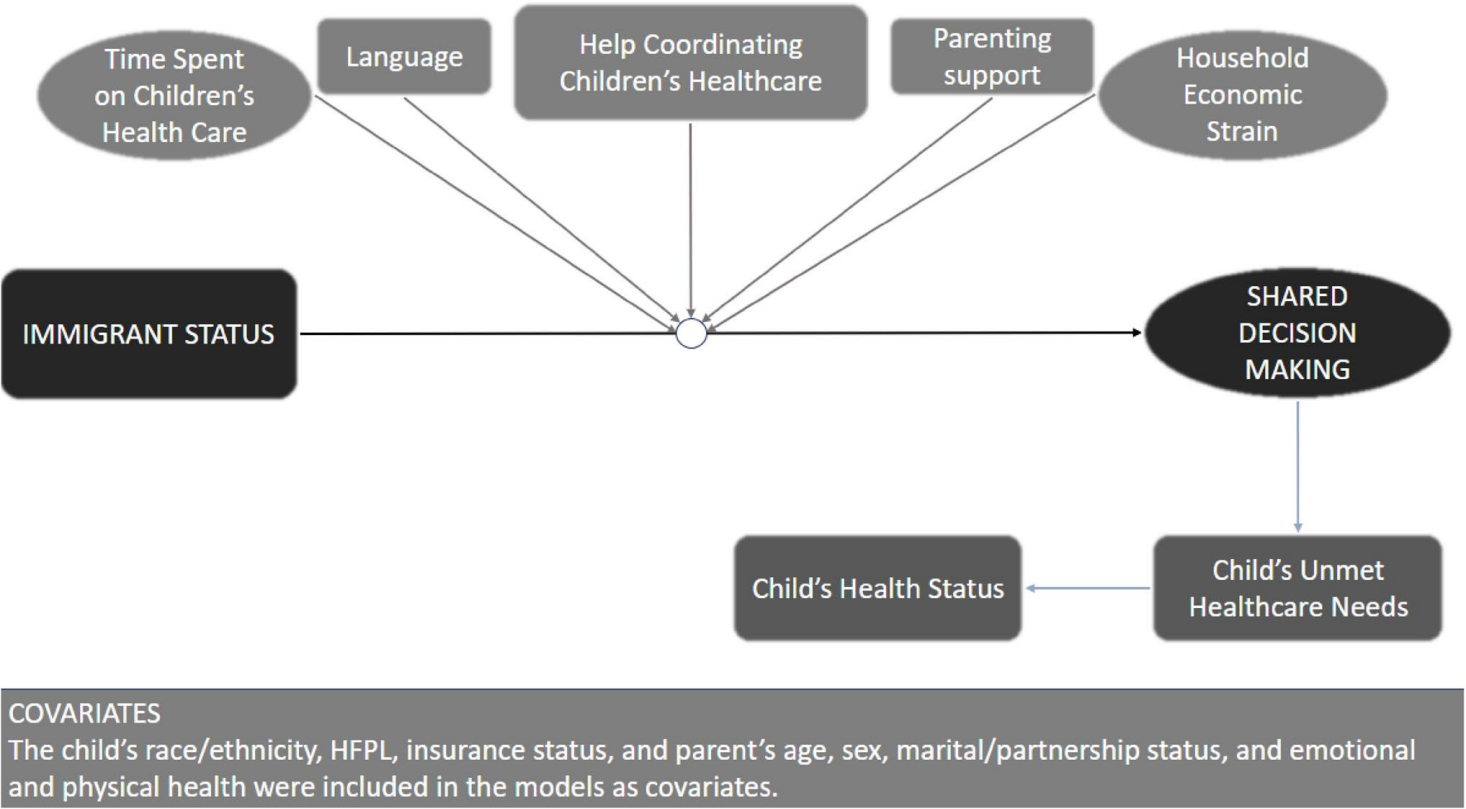
A conceptual model of the associations between immigrant status and shared decision making, and their relation to child health, healthcare outcomes, and respective moderators

Figure 1 presents this study’s conceptual framework. The study examined the association between parents’ immigrant status and their perceptions of SDM in their children’s healthcare. Associations between SDM children’s general health status and unmet healthcare needs were also examined. Lastly, we examined the extent to which this association was moderated by: (1) time spent providing or coordinating their child’s healthcare, (2) household economic strain, (3) parenting support, (4) needing help arranging or coordinating care, and (5) primary household language.

## Methods

### Study Sampling

Data were drawn from the 2021–2022 waves of the National Survey of Children’s Health (NSCH), a cross-sectional, nationally representative study of children’s health and well-being in households with children < 18 years [23]. Data were collected between June 2021 and January 2023 by the U.S. Census Bureau. Utilizing population-level data provides an opportunity to address healthcare outcomes in a representative sample of immigrant parents, and to generalize findings to the broader U.S. population. This cross-sectional study design allowed us to assess population characteristics and prevalence of immigrant parents, SDM, and unmet children’s health care needs, over a two-year period in the U.S. A stratified sampling frame was used to be representative of households with children across the 50 U.S. states and the District of Columbia. Adult respondents completed surveys in English or Spanish, tailored for children in three age groups: 0–5 years, 6–11 years, or 12–17 years. The response rate for the 2021 survey was 40.3% and 39.1% for 2022. Further information on study methodology and confidentiality protection can be accessed at www.childhealthdata.org. Institutional Review Board approval was received from The Pennsylvania State University (protocol #13946); consent to participate was implied.

## Participants

Participants included biological or adoptive parent respondents of children ages 0–17 years living in the same household. Respondents will be referred to as parents from here on. Parents with missing data or reported “No” on whether decisions needed to be made regarding their child’s health were excluded. Lastly, parents were included if they answered *one or more* follow-up questions about SDM. After applying these inclusion and exclusion criteria, 27,082 parents were included in the analytical sample. Outcome variables have different levels of missingness, leading to different sample sizes. However, those cases were kept to not impact weighting. Children’s race/ethnicity, household federal poverty level, and parent education status, have particularly high levels of missingness in the larger sample and were imputed by the National Center for Health Statistics.

## Measures

*Immigrant Status. *Parents reported whether they were born in the U.S. and were categorized as either being U.S.-born or immigrants. Immigrant parents answered a follow-up question: “When did you/this caregiver come to live in the U.S.?” The response was a 4-digit year of migration; time spent in the U.S. was calculated by subtracting the year each parent reported to have arrived in the U.S. from the year the survey was administered. Immigrant parents were categorized into one of the following groups to indicate living in the U.S. between 0 and 4 years, 5–9 years, 10–14 years, or 15 + years, following procedures used by Nwankwo and Wallace [24] and Antecol and Bedard [25].

*Shared Decision Making (SDM)*. SDM is a construct generated from parent responses to three questions. Parents were initially asked whether their children needed decisions to be made regarding their health. If the parent answered “Yes,” three follow-up questions asked the extent to which the child’s doctors/healthcare providers did the following in the past 12 months: discussed a range of healthcare or treatment options, made it easy for parents “to raise concerns or disagree with recommendations,” and worked with the parent on decisions regarding “which healthcare and treatment choices would be best.” Response options were “always,” “usually,” “sometimes,” and “never.” Responses were averaged to create a subscale indicating parents’ reported perceptions of SDM in their child’s healthcare (α = 0.89). The subscale was reverse-coded such that higher scores indicate higher levels of SDM.

*Child Unmet Health Care Needs.* Parents responded yes/no to the question, “During the past 12 months, was there any time when this [child] needed health care but it was not received?”

*Child General Health Status.* Parents were asked, “In general, how would you describe this child’s health?” Response options were “excellent,” “very good,” “good,” “fair,” and “poor.” These response options were reverse coded such that higher numbers indicate better general health status and were then dichotomized to indicate children rated with excellent/very good/good health and poor/fair general health.

*Language.* Parents reported on the primary language used in their home. Options were “English,” “Spanish,” or “Other.” Responses were dichotomized to include “English,” and “Language other than English”.

*Time Spent Providing or Coordinating Health Care.* Parents reported, how many hours in an average week someone in the household spends time “providing healthcare at home for this child” and “arranging or coordinating health or medical care for this child.” Response options for these two questions ranged from “less than 1 hour,” “1–4 hours,” “5–10 hours,” “11 or more hours per week,” or “this child did not need health coordinated [or provided at home] on a weekly basis.” These items were later dichotomized to include 0–4 and 5 + hours per week.

*Support with Child’s Care.* Parents responded yes/no to a question asking whether they felt they “could have used extra help arranging or coordinating this child’s care among the different health care providers or services” in the past 12 months.

*Parenting Support.* Parents responded yes/no to the question, “During the past 12 months, was there someone that you could turn to for day-to-day emotional support with parenting or raising children?”

*Household Economic Strain.* Economic strain was conceptualized using two items. First, parents reported their difficulty in covering “the basics, like food or housing, on your family’s income” since the child was born. Response options were “never,” “rarely,” “somewhat often,” or “very often.” The term *financial strain* was used to conceptualize this question. Second, parents reported whether they had “problems paying for any of this child’s medical or healthcare bills,” during the past 12 months. Response options were “yes” or “no.”

*Sociodemographics.* Parents reported their sex and marital/partnership status, along with their children’s race and ethnicity.

*Covariates.* Household federal poverty level (HFPL) was included as a covariate in all models. Additional covariates included healthcare insurance at the time of the survey (insured/uninsured), parents’ emotional, and physical health status, and parents’ age at interview.

## Statistical Analyses

Analyses were performed using SAS 9.4; PROCSURVEY commands and weights were used to account for the complex survey design. Descriptive analyses were used to describe sample characteristics and to examine frequencies of all main independent and dependent variables, stratified by immigrant status. Adjusted logistic regressions were used to examine significant associations between parent immigrant status and SDM. Lastly, potential moderators of the association between parent immigrant status and SDM were examined. Moderators, including time spent providing or coordinating the child’s healthcare, household economic strain, parenting support, needing extra help arranging or coordinating care, and household language were entered as interaction terms in the multinomial logistic regression models. All moderators were re-coded into binary outcomes before analysis. Moderation effects were identified by determining whether each moderator increased or reduced the odds of SDM among immigrant parents. Logistic regression analyses were conducted sequentially, in a stepwise manner, with each model adjusting for a different set of factors. Model 1 examined unadjusted associations between parent immigrant status and outcome variables. Model 2 adjusted for demographic factors: parent sex, child race, and age. Model 3 adds sociodemographic factors: parent’s marital status, HFPL, and insurance coverage. Model 4 adds parents’ self-reported emotional and physical health.

Given the established association between household poverty and healthcare access [26], and differences in healthcare access by insurance status, logistic regression models were adjusted for HFPL and healthcare coverage. Parent physical health was included as a covariate due to its association with child healthcare access [27, 28]. Preliminary analyses showed that there were significant differences in child race, parent sex, marital status, and mental health by parents’ immigrant status (SI Table A1); these variables were included as covariates in all models.

## Results

### Descriptive Statistics

Descriptives are represented in Table 1. Less than one-fifth (15%) of parents reported being born outside of the U.S. The majority (70.2%) of immigrant parents reported migrating to the U.S. 15 or more years ago. Nearly 6% reported migrating to the U.S. within the last 4 years, 11% within the last 5–9 years, and 13% within the last 10–14 years. The average reported parent age at migration was 20.3 years (SE = 0.6). Parents who reported their sex as female represented 75.2% of the total sample, and 75.8% reported being married at the time of the survey. The average age of U.S.–born parents was 40.7 (SE = 0.1) years and 41.0 years (SE = 0.5) for immigrant parents. About a fifth of immigrant parents (22.7%) reported having a bachelor’s degree, while 19% reported they did not receive a high school diploma or GED. Immigrant parents had lower average HFPL (220.9%, SE = 11.0) as compared to their U.S.-born counterparts (HFPL = 342.9%, SE = 5.6).

**Table 1:** Estimates (weighted and unweighted) of sociodemographic characteristics by immigrant status and total population

Variables	Immigrant Status					
	U.S.-born		Immigrant		Population Estimate (Weighted)	
	*n*	% (SE)	*n*	% (SE)	*N* (in millions)	%
**Number of years in the U.S.**	**23,929**	**85.0 (0.5)**	**2617**	**15.0 (0.5)**	**15.5**	**100.0**
0–4 years	-	-	128	6.1 (1.0)	0.1	0.9
5–9 years	-	-	300	10.5 (1.0)	0.2	1.6
10–14 years	-	-	349	13.2 (1.4)	0.3	2.0
15 years +	-	-	1840	70.2 (1.8)	1.6	10.5
U.S. – born	23,929	100.0 (0.0)	-	-	13.2	85.0
**Relationship to child**						
Biological or adoptive parent	22,064	100.0 (0.0)	2493	100.0 (0.0)	14.0	100.0
**Sex of Primary Caregiver**						
Female	17,990	76.0 (0.5)	1760	70.2 (1.8)	11.7	75.2
Male	5875	24.0 (0.5)	865	29.8 (1.8)	3.8	24.8
**Parent Marital Status**						
Married	18,719	75.7 (0.6)	2083	76.6 (1.8)	11.7	75.8
Not married, living with partner	1158	5.6 (0.3)	135	8.0 (1.3)	0.9	6.0
Never married	1150	6.2 (0.3)	100	3.7 (0.7)	0.9	5.8
Divorced, Separated, or Widowed	2761	12.5 (0.4)	289	11.7 (1.4)	1.9	12.4
**Parent Education Level**						
Less than High School	275	3.3 (0.3)	167	19.0 (2.0)	0.9	5.7
High school graduate or GED	1728	10.2 (0.4)	268	15.6 (1.6)	1.7	11.0
Vocational, trade, business program, or some college, but no degree	3860	17.3 (0.5)	340	12.9 (1.3)	2.6	16.6
Associate degree	2172	8.1 (0.3)	171	5.2 (0.8)	1.2	7.6
Bachelor’s degree	8147	30.9 (0.6)	745	22.7 (1.5)	4.6	29.7
Advanced or Professional Degree (MA, MS, Ph.D., MD, DDS, DVM, JD)	7747	30.2 (0.6)	939	24.7 (1.6)	4.6	29.3
**Financial Strain**						
Very Often	654	3.3 (0.2)	74	3.3 (0.7)	0.5	3.3
Somewhat often	2394	10.9 (0.4)	277	14.6 (1.7)	1.8	11.5
Rarely or never	20,797	85.8 (0.4)	2261	82.0 (1.8)	13.2	85.2
**Problems paying medical bills**						
Yes	3319	18.2 (0.5)	314	23.4 (2.4)	2.2	18.8
No	16,298	81.8 (0.5)	1608	76.6 (2.4)	9.5	81.2
**Hours spent providing health care at home for this child, per week**						
Less than 1 h	3054	48.0 (1.2)	219	37.5 (4.4)	1.7	46.7
1–4 h a week	1669	27.8 (1.1)	145	23.7 (3.6)	1.0	27.3
5–10 h a week	533	9.4 (0.8)	60	14.4 (5.6)	0.4	10.0
11 or more hours a week	829	14.8 (0.9)	109	24.4 (4.4)	0.6	16.0
**Time spent arranging health or medical care, per week (ex, making appointments, locating services)**						
Less than 1 h	4422	65.9 (1.2)	395	63.7 (4.2)	2.5	65.7
1–4 h a week	1656	26.0 (1.1)	162	25.0 (3.6)	1.0	25.9
5–10 h a week	225	3.8 (0.6)	32	4.6 (1.5)	0.1	3.9
11 or more hours a week	181	4.3 (0.6)	27	6.6 (2.0)	0.2	4.6
**Support with Child’s Care**						
Yes	3488	18.8 (0.6)	472	23.4 (1.9)	2.3	19.5
No	15,233	81.2 (0.6)	1514	76.6 (1.9)	9.7	80.5
**Parent’s physical health status**						
Excellent or very good	15,393	63.0 (0.6)	1693	58.4 (2.1)	9.6	62.3
Good health	6689	28.2 (0.6)	700	30.2 (1.9)	4.4	28.5
Fair or Poor	1715	8.8 (0.4)	217	11.4 (1.6)	1.4	9.2
**Parent’s mental/emotional health status**						
Excellent or very good	14,661	61.0 (0.6)	1757	65.5 (2.0)	9.5	61.6
Good health	6863	29.2 (0.6)	645	24.4 (1.8)	4.4	28.5
Fair or Poor	2300	9.8 (0.4)	206	10.1 (1.3)	1.5	9.8
**Someone to turn to for parenting support**						
Yes	20,747	87.0 (0.4)	1696	55.7 (2.1)	12.7	82.3
No	3050	13.0 (0.4)	901	44.3 (2.1)	2.7	17.7
**Language**						
English	23,642	98.8 (0.2)	1652	52.0 (2.1)	14.2	91.8
Spanish	111	0.8 (0.2)	468	32.8 (2.1)	0.9	5.6
Other	78	0.4 (0.1)	480	15.2 (1.3)	0.4	2.6
	*n* = 23,929			*n* = 2630		
	Median	95% CI	SE	Median	95% CI	SE
**Shared Decision Making**	3.7	(3.7, 3.7)	0.0	3.6	(3.4, 3.9)	0.1
Age*	40.7	(40.4, 41.0)	0.1	41.0	(40.2, 42.0)	0.5
Federal Poverty Level*	342.9	(331.9, 354.0)	5.6	220.9	(199.3, 242.5)	11.0
Migration Statistics**				*n* = 2630		
Age at Migration (Years)	**-**	**-**	**-**	20.3	(19.0, 21.5)	0.6
Year of U.S. Migration	**-**	**-**	**-**	2000.2	(1999.2, 2001.2)	0.5

Bold indicates construct names or the general category that a variable would fall under

*Age of the parent completing the survey and their federal poverty level

**Calculated age at migration and the year parents migrated is reported for immigrant parents only

Among immigrant parents, 3.3% reported experiencing financial strain “very often.” On average, 23.4% of immigrant and 18.2% of U.S.-born parents reported experiencing problems paying their child’s medical bills. Over one-third of immigrant parents (38.8%) and one-quarter of U.S.-born parents (24.2%) reported spending 5 + hours a week, on average, providing health care at home for their child. Roughly one-tenth of immigrant (11.2%) and U.S.-born (8.1%) parents reported spending 5 + hours a week, on average, arranging healthcare services. Most immigrant (76.6%) and U.S.-born parents (81.2%) reported that they did not need extra help coordinating care for their child. More U.S.-born parents reported they had someone to turn to for parenting support (87%) as compared to immigrant parents (55.7%). The majority of immigrant (58.4%) and U.S.-born (63%) parents reported themselves to be in excellent or very good physical health. Most immigrant parents (65.5%) and U.S.-born parents (61%) reported being in excellent or very good mental or emotional health. English was reported as the primary household language by 52% of immigrant parents and 98.8% of U.S.-born parents.

## Associations between Parent Immigrant Status, SDM, and Child Health Outcomes

Table 2 presents stepwise analyses of the associations between immigrant status and reports of SDM, child health status, and unmet healthcare needs. Results showed that compared to U.S.-born parents, immigrant parents had significantly higher odds of reporting lower levels of SDM, after adjusting for demographic, socioeconomic, and parent health factors (*p* < 0.01). Compared to U.S.-born parents, immigrant parents also had higher odds of reporting that their child was in fair or poor health (*p* < 0.01). There were no significant associations between immigrant status and reports of children’s unmet healthcare needs (Table 2).

**Table 2:** Associations between parent immigrant status and SDM, child health status, and unmet health care needs

Variable	Model 1: Unadjusted			Model 2: Demographic Factors^a^			Model 3: Socioeconomic Factors^b^			Model 4: Health of Parent^c^		
	*n*	OR (95%CI)	*p*-value	*n*	OR (95%CI)	*p*-value	*n*	OR (95%CI)	*p*-value	*n*	OR (95%CI)	*p*-value
Shared Decision Making	26,506	**1.8 (1.6**,** 2.2)**	**< 0.01**	26,378	**1.8 (1.5**,** 2.2)**	**< 0.01**	24,251	**1.7 (1.4**,** 2.0)**	**< 0.01**	26,060	**1.7 (1.5**,** 2.1)**	**< 0.01**
Child health status	26,495			26,364			26,171			26,041		
*Fair or poor health*		**1.7 (1.3**,** 2.1)**	**< 0.01**		**1.7 (1.4**,** 2.1)**	**< 0.01**		**1.4 (1.2**,** 1.8)**	**< 0.01**		**1.5 (1.2**,** 1.9)**	**< 0.01**
Unmet healthcare needs	26,504			26,374			26,181			26,051		
*Yes*		1.2 (0.9, 1.6)	0.19		1.2 (0.9, 1.6)	0.21		1.0 (0.7, 1.4)	0.97		1.1 (0.8, 1.5)	0.63

Note: Immigrant parents are compared to the reference group, U.S.-born parents

^a^Model 2 adjusted for: sex of parent, race of child, parent age

^b^Model 3 adjusted for: sex of parent, race of child, parent age, parent’s marital status, federal poverty level, child insurance coverage

^c^Model 4 adjusted for: sex of parent, race of child, parent age, parent’s marital status, federal poverty level, child insurance coverage, parent’s mental and physical health

Associations significant at α = 0.05 are represented in **bold**

Table 3 presents associations between immigrant status and potential moderators. Compared to U.S.-born parents, immigrant parents reported significantly higher odds of spending 5 + hours a week *providing care* at home for their child after adjusting for demographic, socioeconomic, and parental health variables (*p* < 0.01). Immigrant parents, as compared to U.S.-born parents, also had significantly higher odds of reporting they needed extra help with care coordination (*p* = 0.04).

**Table 3:** Associations with and moderating effects of time spent on healthcare, economic strain, household language, and parenting support on the association between immigrant status and SDM

**Variable**	Model 1: Unadjusted			Model 2: Demographic Factors^a^			Model 3: Socioeconomic Factors^b^			Model 4: Health of Parent^c^			Moderation Effect SDM		
	n	OR (95%CI)	p-value	n	aOR (95%CI)	p-value	n	aOR (95%CI)	p-value	n	aOR (95%CI)	p-value	n	aOR (95%CI)	p-value
**Time Spent on Healthcare**															
Time spent arranging care	7100			7071			7026			6984			6979		
*5 + hours a week*		1.4 (0.8, 2.5)	0.17		1.5 (0.9, 2.5)	0.16		1.4 (0.8, 2.4)	0.28		1.4 (0.8, 2.4)	0.28		0.9 (0.4, 2.0)	0.84
Time spent providing care at home	6618			6592			6553			6512			6505		
*5 + hours a week*		**2.0 (1.2**,** 3.2)**	**< 0.01**		**2.0 (1.2**,** 3.2)**	**< 0.01**		**1.8 (1.1**,** 2.9)**	**0.01**		**1.8 (1.2**,** 2.8)**	**< 0.01**		1.1 (0.5, 2.5)	0.77
**Support with Child’s Care**															
Needed extra help with care coordination	20,707			20,602			20,460			20,363			20,335		
*Yes*		**1.3 (1.1**,** 1.6)**	**0.01**		**1.3 (1.1**,** 1.7)**	**0.01**		1.2 (1.0, 1.5)	0.09		**1.3 (1.0**,** 1.6)**	**0.04**		**3.2 (2.1**,** 4.9)**	**< 0.01**
**Economic Strain**															
Financial Strain	26,457			26,338			26,163			26,033			25,994		
*Very Often*		**0.8 (0.6**,** 1.0)**	**0.04**		**0.7 (0.6**,** 1.0)**	**0.02**		0.9 (0.7, 1.1)	0.33		0.9 (0.7, 1.1)	0.26		1.5 (0.9, 2.3)	0.09
Problems paying medical bills	21,539			21,461			21,335			21,240			21,215		
*Yes*		**1.4 (1.1**,** 1.8)**	**0.02**		**1.5 (1.1**,** 1.9)**	**< 0.01**		1.2 (0.9, 1.6)	0.14		1.3 (1.0, 1.7)	0.67		0.8 (0.5, 1.3)	0.36
**Household Language**															
	26,431			26,305			26,111			25,982			25,940		
*English*		**0.01 (0.01**,** 0.02)**	**< 0.01**		**0.01 (0.01**,** 0.02)**	**< 0.01**		**0.01 (0.01**,** 0.02)**	**< 0.01**		**0.01 (0.01**,** 0.02)**	**< 0.01**		1.4 (1.0, 1.9)	0.06
**Parenting Support**															
Someone to turn to for emotional and parenting support	26,394			26,273			26,103			25,976			25,941		
*No*		**5.3 (4.4**,** 6.4)**	**< 0.01**		**5.2 (4.4**,** 6.3)**	**< 0.01**		**4.7 (3.9**,** 5.7)**	**0.01**		**5.0 (4.1**,** 6.1)**	**< 0.01**		1.3 (1.0, 1.8)	0.09

Note: In Models 1–4, immigrant parents were compared to the reference group, U.S.-born parents

Moderation effect comparisons were made *within* immigrant parents. Parents who reported: spending 5 + hours a week on healthcare, needing extra help with care coordination, experiencing financial strain somewhat or very often, having problems paying medical bills, their primary household language to be a language other than English, and not having parental support were compared to the reference groups, parents who reported spending 0–4 h a week on healthcare, not needing extra help with care coordination, experiencing financial strain rarely or never, not experiencing problems paying medical bills, English as their primary household language, and having parental support, respectively

^a^Model 2 adjusted for: sex of parent, race of child, parent age

^b^Model 3 adjusted for: sex of parent, race of child, parent age, parent’s marital status, federal poverty level, child insurance coverage

^c^Model 4 adjusted for: sex of parent, race of child, parent age, parent’s marital status, federal poverty level, child insurance coverage, parent’s mental and physical health

Associations significant at α = 0.05 are represented in **bold**

Compared to U.S.-born parents, immigrant parents had significantly lower odds of reporting that they experience financial strain “Very Often” in the model adjusting for demographic factors (*p* = 0.02). This association was no longer significant after adjusting for socioeconomic or parent health factors. After adjusting for demographic factors, immigrant parents had significantly higher odds of reporting they experienced problems paying for their child’s healthcare (*p* < 0.01) compared to U.S.-born parents. This association was no longer significant after adjusting for socioeconomic or parent health factors. Immigrant parents showed significantly lower odds of reporting “English” as their primary household language, as compared to their U.S.-born counterparts (*p* < 0.01). Lastly, immigrant parents, compared to U.S.-born parents, showed significantly higher odds of reporting that they did *not* have someone to turn to for parenting support (*p* < 0.01), after adjusting for all demographic, socioeconomic, and parent health variables.

## Moderation Effects

Table 3 also presents estimates for moderation effects. We examined whether moderating variables emerged as risk or protective factors for SDM among immigrant parents. Thus, these findings can be interpreted as the extent to which the moderating variable increased (OR > 1) or decreased (OR < 1) the odds of parents reporting lower levels of SDM. Compared to their counterparts, immigrant parents who reported needing extra help with care coordination had significantly higher odds of reporting lower levels of SDM (*p* < 0.01). No other variables emerged as significant moderators in the association between immigrant status and SDM.

## Discussion

Findings from this study provide evidence of inequities in SDM among immigrant parents. To the best of our knowledge, this is the first study to examine the association between parent immigrant status and SDM with children’s healthcare providers on children’s health outcomes in a nationally representative survey. Immigrant parents had significantly higher odds of reporting (1) lower levels of SDM, (2) higher odds of reporting that their children were in fair or poor health, and (3) that they spent more time providing healthcare at home, compared to U.S.-born parents. A previous study showed that having someone more knowledgeable about the healthcare system assist with appointments increased informed decision-making [29]; however, this was examined among African immigrants. We add to these findings by showing that when immigrant parents felt they had sufficient assistance with their child’s healthcare coordination (i.e., between different healthcare providers), they reported higher levels of SDM, compared to immigrant parents who reported needing extra help with care coordination.

We did not find evidence for emotional support with parenting as a significant moderator of the association between immigrant status and SDM. This finding contradicts what is in the literature, as previous studies show that parenting social support, (i.e., social support from a family/friend) is a facilitator in the SDM process [30, 31], and critical in establishing health-seeking behaviors [29, 32, 33]. However, compared to other sources of support, parental *emotional* support is a more general form of parenting support, which may explain why it was a less salient moderator, compared to support with care coordination. Together, findings from the present study suggest that programs that provide support with care coordination may be important in improving SDM among immigrant parents, which may reduce inequities in SDM due to immigrant status.

A systematic review found that common barriers to SDM in pediatric clinics include environmental factors, such as time and access to resources [30]. Individuals who resettle to the U.S. have been shown to struggle with seeking help for medical conditions [5, 34]. A previous study found that African mothers would seek medical help only when they believed the benefits outweighed the barriers, including health literacy and the complexity of the healthcare system [29]. As a result, patients who do not receive the support they need in utilizing the healthcare system may be less likely to engage in the SDM process. This may have substantial effects on patient’s healthcare quality and health outcomes [30, 35]. Few studies have examined the effects of parents’ SDM in children’s health care on children’s health outcomes; we are not aware of any such published studies in immigrant parents.

### Study Strengths and Limitations

This study included data from a large, nationally representative sample, allowing for the generalization of findings to U.S. households with children. However, this study is not without limitations. The cross-sectional design limits our ability to infer causality. In addition, data collection took place between June 2021 and January 2023, overlapping with the COVID-19 pandemic. Associations reported in this sample may be characteristically different from what may have been reported before the pandemic. Furthermore, the length of the NSCH may have contributed to response bias. The NSCH also may not represent families who do not speak English or Spanish, as the surveys were only offered in those languages. This study may not be fully representative of immigrant parents because (1) our operationalization of “immigrant status” is limited to what was available in the NSCH, which lacks details accounting for residency, visa status, or citizenship, and (2) most immigrant parents in this study reported living in the U.S. for 15 + years making them differ from more recent immigrants as a result of acculturation [25, 36] and insurance status [37]. Future studies should include more detailed emigration data to allow for a more nuanced understanding of the association between SDM and immigrant status.

### Conclusions and Implications for Practice

Findings from this study add to the SDM literature by highlighting the need for SDM and support in coordinating children’s health care in immigrant parents. Doing so may improve patient health outcomes and increase healthcare utilization [35, 37]. Culturally appropriate, community-based partnerships between parents and healthcare providers have been shown to assist immigrants in navigating the healthcare system [34, 38, 39]. Future studies should investigate additional factors that may increase SDM in immigrant parents such as using more detailed information on immigrant status and experiences navigating the health care system for children.

## Supplementary Information

Below is the link to the electronic supplementary material.

Supplementary material 1 (DOCX 14.7 kb)

## References

[1] U.S. Census Bureau. Selected Characteristics of the Native and Foreign-Born Populations [Internet]. American Community Survey; 2022 [cited 2025 Jan 15]. Available from: https://data.census.gov/table/ACSST1Y2022.S0501?q=Native and Foreign-Born&g = 010XX00US&y = 2022.

[2] U.S. Census Bureau USD of C. Selected Characteristics of the Native and Foreign-Born Populations [Internet]. American Community Survey; 2023 [cited 2025 Jan 15]. Available from: https://data.census.gov/table/ACSST1Y2023.S0501?q=Native and Foreign-Born&g = 010XX00US&y = 2023.

[3] Betz CL. Immigrant Children: Unmet Needs and a Myriad of Nursing Concerns. J Pediatr Nurs [Internet]. 2008 [cited 2023 Feb 20];23:157–60. Available from: https://linkinghub.elsevier.com/retrieve/pii/S0882596308001188 10.1016/j.pedn.2008.02.001 18492544

[4] U.S. Department of Health and Human Services. Health resources and services administration, maternal and child health bureau. child health USA 2011. Maryland: U.S. Department of Health and Human Services,: Rockville; 2011.

[5] Huang ZJ, Yu SM, Ledsky R. Health status and health service access and use among children in U.S. immigrant families. Am J Public Health. 2006;96:634–40. 16507736 10.2105/AJPH.2004.049791 PMC1470552

[6] Pumariega AJ, Rothe E, Pumariega JB. Mental health of immigrants and refugees. Community Ment Health J. 2005;41:581–97. 10.1007/s10597-005-6363-1. 16142540 10.1007/s10597-005-6363-1

[7] Charles C, Gafni A, Whelan T. Shared decision-making in the medical encounter: What does it mean? (or it takes at least two to tango). Soc Sci Med [Internet]. 1997 [cited 2023 Jun 30];44:681–92. Available from: https://linkinghub.elsevier.com/retrieve/pii/S0277953696002213 10.1016/s0277-9536(96)00221-3 9032835

[8] Waldron T, Carr T, McMullen L, Westhorp G, Duncan V, Neufeld S-M, et al. Development of a program theory for shared decision-making: a realist synthesis. BMC Health Serv Res. 2020;20: 59. 10.1186/s12913-019-4649-1. 31973754 10.1186/s12913-019-4649-1 PMC6979294

[9] Clever SL, Ford DE, Rubenstein LV, Rost KM, Meredith LS, Sherbourne CD et al. Primary Care Patients’ Involvement in Decision-Making Is Associated With Improvement in Depression. Med Care [Internet]. 2006 [cited 2023 Jul 23];44:398–405. Available from: https://journals.lww.com/00005650-200605000-00003 10.1097/01.mlr.0000208117.15531.da 16641657

[10] Fiks AG, Mayne SL, Karavite DJ, Suh A, O’Hara R, Localio AR et al. Parent-Reported Outcomes of a Shared Decision-Making Portal in Asthma: A Practice-Based RCT. Pediatrics [Internet]. 2015 [cited 2023 Jun 30];135:e965–73. Available from: https://publications.aap.org/pediatrics/article/135/4/e965/33582/Parent-Reported-Outcomes-of-a-Shared-Decision 10.1542/peds.2014-3167 PMC4379463 25755233

[11] Kaplan SH, Greenfield S, Ware JEJ. Assessing the Effects of Physician-Patient Interactions on the Outcomes of Chronic Disease. Med Care [Internet]. 1989;27. Available from: https://journals.lww.com/lww-medicalcare/Fulltext/1989/03001/Assessing_the_Effects_of_Physician_Patient.10.aspx 10.1097/00005650-198903001-00010 2646486

[12] Staiti AB, Hurley RE, Katz A. Stretching the safety net to serve undocumented immigrants: community responses to health needs. Issue Brief Cent Stud Health Syst Change. 2006;1–4. 16506345

[13] Alegría M, Sribney W, Perez D, Laderman M, Keefe K. The role of patient activation on patient–provider communication and quality of care for US and foreign born Latino patients. J Gen Intern Med. 2009;24(S3):534–41. 10.1007/s11606-009-1074-x. 19842003 10.1007/s11606-009-1074-x PMC2764038

[14] Cooper-Patrick L. Race, gender, and partnership in the patient-physician relationship. JAMA. 1999;282: 583. 10.1001/jama.282.6.583. 10450723 10.1001/jama.282.6.583

[15] Jolles MP, Lee P-J, Javier JR. Shared decision-making and parental experiences with health services to meet their child’s special health care needs: Racial and ethnic disparities. Patient Educ Couns [Internet]. 2018 [cited 2023 Feb 20];101:1753–60. Available from: https://linkinghub.elsevier.com/retrieve/pii/S0738399118302696 10.1016/j.pec.2018.05.022 29884531

[16] Chipman SA, Meagher K, Barwise AK. A public health ethics framework for populations with limited English proficiency. Am J Bioeth. 2023;(11):65. https://www.tandfonline.com/doi/full/10.1080/15265161.2023.2224263 10.1080/15265161.2023.2224263 37379053

[17] Espinoza Suarez NR, Urtecho M, Nyquist CA, Jaramillo C, Yeow M-E, Thorsteinsdottir B et al. Consequences of suboptimal communication for patients with limited English proficiency in the intensive care unit and suggestions for a way forward: A qualitative study of healthcare team perceptions. J Crit Care [Internet]. 2021 [cited 2023 Jul 23];61:247–51. Available from: https://linkinghub.elsevier.com/retrieve/pii/S0883944120307334 10.1016/j.jcrc.2020.10.012 PMC8423101 33221592

[18] Ferguson WJ, Candib LM. Culture, language, and the doctor-patient relationship. Fam Med. 2002;34:353–61. 12038717

[19] Palmer NRA, Kent EE, Forsythe LP, Arora NK, Rowland JH, Aziz NM et al. Racial and Ethnic Disparities in Patient-Provider Communication, Quality-of-Care Ratings, and Patient Activation Among Long-Term Cancer Survivors. J Clin Oncol [Internet]. 2014 [cited 2023 Feb 20];32:4087–94. Available from: 10.1200/JCO.2014.55.5060 10.1200/JCO.2014.55.5060 PMC4265119 25403220

[20] Thomas KC, Stein GL, Williams CS, Jolles MP, Sleath BL, Martinez M, et al. Fostering activation among Latino parents of children with mental health needs: an RCT. Psychiatr Serv. 2017;68:1068–75. 10.1176/appi.ps.201600366. 28566024 10.1176/appi.ps.201600366

[21] Dobler CC, Spencer-Bonilla G, Gionfriddo MR, Brito JP. Shared Decision Making in Immigrant Patients. Cureus [Internet]. 2017 [cited 2023 Feb 20]; Available from: http://www.cureus.com/articles/7088-shared-decision-making-in-immigrant-patients 10.7759/cureus.1461 PMC5595268 28936373

[22] Wood F, Phillips K, Edwards A, Elwyn G. Working with interpreters: The challenges of introducing Option Grid patient decision aids. Patient Educ Couns [Internet]. 2017 [cited 2023 Feb 20];100:456–64. Available from: https://linkinghub.elsevier.com/retrieve/pii/S073839911630444X 10.1016/j.pec.2016.09.016 27745941

[23] Child and Adolescent Health Measurement Initiative (CAHMI). Maternal and Child Health Bureau (MCHB). Health Resources and Services Administration (HRSA); 2024. [05/08/24] from childhealthdata.org. 2021–2022 National Survey of Children’s Health (2 years combined), [(SAS/SPSS/Stata)] dataset. Data Resource Center for Child and Adolescent Health supported by Cooperative Agreement U59MC27866 from the U.S. Department of Health and Human Services,.

[24] Nwankwo EM, Wallace SP. Duration of United States residence and self-reported health among African-born immigrant adults. J Immigr Minor Health. 2021;23(4):773–83. 10.1007/s10903-020-01073-8. 32845410 10.1007/s10903-020-01073-8 PMC8074510

[25] Antecol H, Bedard K. Unhealthy assimilation: Why do immigrants converge to American health status levels? Demography [Internet]. 2006 [cited 2024 Jun 2];43:337–60. Available from: https://read.dukeupress.edu/demography/article/43/2/337/170120/Unhealthy-assimilation-Why-do-immigrants-converge 10.1353/dem.2006.0011 16889132

[26] Larson K, Halfon N. Family income gradients in the health and health care access of US children. Matern Child Health J. 2010;14(3):332–42. 10.1007/s10995-009-0477-y. 19499315 10.1007/s10995-009-0477-y PMC2862175

[27] Riportella-Muller R, Selby-Harrington ML, Richardson LA, Donat PL, Luchok KJ, Quade D. Barriers to the use of preventive health care services for children. Public Health Rep Wash DC 1974. 1996;111:71–7. PMC1381746 8610196

[28] Staff I of, Staff M, Edmunds NRCM. America’s Children: Health Insurance and Access to Care. Washington: National Academies Press; 1998. 25101405

[29] Tefera GM. Understanding access, and utilization of healthcare services among African immigrant women in the United States. : The application of health belief model. Appl Res Qual Life. 2024;19:1097–115. 10.1007/s11482-024-10283-3.

[30] Boland L, Graham ID, Légaré F, Lewis K, Jull J, Shephard A, et al. Barriers and facilitators of pediatric shared decision-making: a systematic review. Implement Sci. 2019;14: 7. 30658670 10.1186/s13012-018-0851-5 PMC6339273

[31] Brabers AEM, De Jong JD, Groenewegen PP, Van Dijk L. Social support plays a role in the attitude that people have towards taking an active role in medical decision-making. BMC Health Serv Res. 2016;16: 502. 10.1186/s12913-016-1767-x. 27655113 10.1186/s12913-016-1767-x PMC5031318

[32] Campisi C, Pham D, Rapoport E, Adesman A. Parenting, stress. Community support, and unmet health care needs of children in the US. Matern Child Health J. 2024;28:1010–9. 10.1007/s10995-024-03912-8. 38353888 10.1007/s10995-024-03912-8

[33] Raphael JL, Zhang Y, Liu H, Giardino AP. Parenting stress in US families: implications for paediatric healthcare utilization. Child Care Health Dev. 2010;36(2):216–24. 10.1111/j.1365-2214.2009.01052.x. 20047600 10.1111/j.1365-2214.2009.01052.x

[34] Alwan RM, Schumacher DJ, Cicek-Okay S, Jernigan S, Beydoun A, Salem T, et al. Beliefs, perceptions, and behaviors impacting healthcare utilization of Syrian refugee children. PLoS One. 2020;15:e0237081. 32764783 10.1371/journal.pone.0237081 PMC7413502

[35] Hughes TM, Merath K, Chen Q, Sun S, Palmer E, Idrees JJ, et al. Association of shared decision-making on patient-reported health outcomes and healthcare utilization. Am J Surg. 2018;216:7–12. 29395026 10.1016/j.amjsurg.2018.01.011

[36] The Integration of Immigrants into American Society [Internet], Washington DC. National Academies Press; 2015 [cited 2024 Jul 10]. p. 21746. Available from: http://www.nap.edu/catalog/21746

[37] Thamer M, Richard C, Casebeer AW, Ray NF. Health insurance coverage among foreign-born US residents: the impact of race, ethnicity, and length of residence. Am J Public Health. 1997;87:96–102. 10.2105/AJPH.87.1.96. 9065235 10.2105/ajph.87.1.96 PMC1380772

[38] Linton JM, Stockton MP, Andrade B, Daniel S. Integrating Parenting Support Within and Beyond the Pediatric Medical Home. Glob Pediatr Health [Internet]. 2018 [cited 2025 Jan 30];5:2333794X18769819. Available from: https://www.ncbi.nlm.nih.gov/pmc/articles/PMC5946342/10.1177/2333794X18769819 PMC5946342 29761138

[39] Shah MK, Wyatt LC, Gibbs-Tewary C, Zanowiak JM, Mammen S, Islam NA, Culturally Adapted. Telehealth, Community Health Worker Intervention on Blood Pressure Control among South Asian Immigrants with Type II Diabetes: Results from the DREAM Atlanta Intervention. J Gen Intern Med [Internet]. 2024 [cited 2025 Jan 30];39:529–39. Available from: 10.1007/s11606-023-08443-6 10.1007/s11606-023-08443-6 PMC10973296 37845588

