# Supplementary material for: Shared Decision-Making in Children’s Healthcare by Parents’ Immigrant Status: Findings from the 2021–2022 U.S. National Survey of Children’s Health
Source: J Immigr Minor Health. 2025 Oct 17;28(1):176–86. doi: 10.1007/s10903-025-01771-1 (PMC12882855; doi:10.1007/s10903-025-01771-1)
Supplement: Supplementary file 1 — Supplementary material 1 (DOCX 14.7 kb) [file 10903_2025_1771_MOESM1_ESM.docx]

**Supplemental Information**

| **Table A1:** Associations with Nativity | | | |
| --- | --- | --- | --- |
| **VARIABLES** | **n** | **OR** | **95% CI** |
| **Shared Decision Making** | 26506 |  |  |
| Low |  | **1.8** | **(1.6, 2.1)** |
| **Unmet Health Care Needs** | 26504 |  |  |
| Yes |  | 1.2 | (0.9, 1.6) |
| **SOCIODEMOGRAPHICS** |  |  |  |
| **Race** | 26559 |  |  |
| Hispanic |  | **2.7** | **(2.1, 3.3)** |
| **Current Coverage** | 26511 |  |  |
| Yes |  | **2.7** | **(1.7, 4.4)** |
| **Sex of parent** | 26490 |  |  |
| Male |  | **1.3** | **(1.1, 1.6)** |
| **Parent Marital Status** | 26395 |  |  |
| Married |  | **1.1** | **(0.9, 1.3)** |
| **PARENT HEALTH** |  |  |  |
| **Parent Physical Health** | 26407 |  |  |
| Excellent or very good health |  | 0.8 | (0.7, 1.0) |
| **Parent Mental Health** | 26432 |  |  |
| Excellent or very good mental health |  | **1.2** | **(1.0, 1.4)** |
| **Physical health of secondary caregiver** | 22706 |  |  |
| Excellent or very good health |  | 0.9 | (0.7, 1.1) |
| **ECONOMIC STRAIN** |  |  |  |
| **Problems paying for medical bills** | 21539 |  |  |
| Yes |  | **1.4** | **(1.1, 1.8)** |
| **Financial Strain** | 26457 |  |  |
| Very often |  | **1.3** | **(1.0, 1.7)** |
| **TIME SPENT ON HEALTHCARE** |  |  |  |
| **Time spent arranging care at home** | 7100 | 1.4 | (0.8, 2.5) |
| 5+ hours a week |  |  |  |
| **Time spent providing care at home** | 6618 |  |  |
| 5+ hours a week |  | **2.0** | **(1.2, 3.2)** |
| **Extra help needed to coordinate care** | 20707 |  |  |
| Yes |  | **1.3** | **(1.1, 1.6)** |
| **PARENTING SUPPORT** |  |  |  |
| **Someone to turn to for emotional and parenting support** | 21539 |  |  |
| Yes |  | **1.4** | **(1.1, 1.8)** |
